# Sharing Online Health Information With Physicians: Understanding the Associations Among Patient Characteristics, Directness of Sharing, and Physician-Patient Relationship

**DOI:** 10.3389/fpsyg.2022.839723

**Published:** 2022-03-30

**Authors:** Siyue Li, Kexin Wang

**Affiliations:** College of Media and International Culture, Zhejiang University, Hangzhou, China

**Keywords:** online health information sharing, communication apprehension, eHealth literacy, physician patient communication, patient satisfaction

## Abstract

Patients increasingly share online health information with their physicians. However, few studies have investigated factors that may facilitate or inhibit such sharing and subsequent impact on physician-patient relationship. This study conducted a cross-sectional survey among 818 Chinese patients to examine if two patient characteristics -communication apprehension and eHealth literacy- influence their ways of sharing online health information with physicians and subsequently impact physician-patient relationship. The results showed that a majority of surveyed participants searched health information online, and about half of them used such information during their doctor visits. Less apprehensive patients tend to share the information with their physicians more directly, which can positively affect perceived physician reactions and patient satisfaction. eHealth literacy, however, is not found to be associated with patients’ sharing of online information with physicians. This study underscores the importance of identifying patient characteristic’s role in patient-physician interaction.

## Introduction

The rise of the Internet has significantly changed the ways through which that patients acquire health information (Wong and Cheung, 2019). Besides passively receiving information from physicians, patients nowadays can actively search for health information *via* different online outlets (Wang et al., 2020). The easy access of online health information, to a certain extent, has shifted the power in physician-patient relationship. Patients become more informed about their health and take a more active role in health-decision making.

Health information acquired online may serve as a double-edged sword for patients. Although patients can educate themselves *via* online platforms, they may also receive misleading and even false information online (Suarez-Lledo and Alvarez-Galvez, 2021). One way to reduce the confusion over online health information is through open discussion with physicians, yet some research shows that patients do not always share online health information with their physicians (Matusitz and Spear, 2015). Patients are worried that physicians may feel challenged by the shared information, potentially leading to a relational damage with their physicians. However, the other line of research shows that many patients still choose to strategically reveal such information during doctor visits (Tan and Goonawardene, 2017). The conflicting findings revealed in extant literature might be attributed in part to the complex construct of online health information sharing with physicians. Rather than dichotomizing sharing behaviors into yes and no, patients can differ in their extent of sharing online health information with physicians. Past research showed that some patients explicitly shared the information with their physicians and others covertly compared online information with the information provided by physicians (Sommerhalder et al., 2009). The various ways of information sharing thus differ along the spectrum of directness. Directness of online health information sharing is defined as the extent to which patients explicitly share health information found online with their physicians. This study attempts to examine this overlooked aspect–directness of online health information sharing with physicians–in a nuanced way by specifically focusing on its antecedents and relational outcomes.

Apart from past research which heavily examined situational factors and physician attributes (Tan and Goonawardene, 2017), this study highlights the importance of patient characteristics in their influence on sharing online health information with physicians. Specifically, this study examines two characteristics of patients—communication apprehension and eHealth literacy—in online health information sharing with physicians. Communication apprehension, characterized by anxiety in social interactions (McCroskey, 1984), is highly relevant to the communication activity of online health information sharing. Patients varying in this characteristic are expected to differ in information sharing behaviors with their physicians (Booth-Butterfield et al., 1997), which may subsequently influence physician-patient relationship as well as treatment outcomes (Perrault and Silk, 2015). Given the importance of communication apprehension in health communication, we need to closely look into the role of communication apprehension in sharing Internet search with physicians and shaping physician-patient relationship. In addition, eHealth literacy may serve as a motivation to share online health information with physicians (Briones, 2015). eHealth literacy pertains to patients’ abilities to acquire and evaluate online health information. As a well-established construct in health communication, eHealth literacy has been mostly examined in online health information seeking, a solidary activity requiring minimal social interaction (Chang et al., 2015). Its role in health communication, particularly online health information sharing, has been largely overlooked. To fill the gap, this study examines the impact of eHealth literacy on physician-patient interaction.

Besides patient characteristics, this study strives to examine relational outcomes associated with the directness of online health information sharing. Although physician-patient relationship has been a long-standing topic in health communication, the specific ways of sharing online health information with physicians have yet to be linked to the relational outcomes. To fill the gap, this study investigates if and how the directness of online information sharing affects patients’ perceived physician reactions and thereby patient satisfaction.

## Sharing Online Health Information With Physicians

An increase in online health information seeking is likely to lead to a rise in information sharing during doctor visits. As Hu et al. (2012) suggested, patients search for health information online to get prepared for their upcoming doctor visits. Their study found that more than half of the participants planned to ask their physicians questions about the information they found online and roughly one-third of the participants indicated that they had printed out online information to share with the doctors. At the same time, another body of literature acknowledged patients’ concerns about sharing online health information and asking questions during doctor visits, due to a fear of challenging the physicians’ authority (Matusitz and Spear, 2015).

To understand the extent to which patients would share online health information during doctor visits, past research examined facilitators and barriers to reveal online information with physicians (Tan and Goonawardene, 2017). Factors that motivate patients to share online information include but are not limited to having a family member accompanied during a doctor visit, physicians encouraging patients to discuss online search, and patients feeling a strong need to check online information with physicians (Stevenson et al., 2007; Silver, 2015). In contrast, pre-established view of the physician-patient relationship, perceived authority of physicians, and perceived embarrassment while asking questions pose obstacles to information sharing with physicians (Hart et al., 2004; Silver, 2015).

Research has also suggested that patients adopt different strategies to use and reveal online information during doctor visits (Sommerhalder et al., 2009; Wong and Cheung, 2019). For instance, while some patients choose to silently verify online findings without asking any questions, others may explicitly ask questions or even show physicians their online findings in person (Tan and Goonawardene, 2017). Although the directness of online health information sharing with physicians has not been explicitly examined in prior research, these identified strategies can be differentiated along the spectrum of directness, with one end of not mentioning the Internet search and the other end of directly showing online information to physicians. Given the large variation in using online health information, this study is interested in whether communication apprehension and eHealth literacy may serve as facilitators or deterrents of sharing online information with physicians.

## Communication Apprehension

Communication apprehension is conceptualized as “an individual’s level of fear or anxiety associated with either real or anticipated communication with another person or persons” (McCroskey, 1984, p.13). Past research found that highly apprehensive patients would feel a sense of powerlessness during doctor visits and feel reluctant to communicate with their physicians (Wheeless, 1984). A lack of communication between patients and physicians may lead to negative consequences on relationships and health outcomes (Perrault and Silk, 2015).

To date, research in health communication has only broadly assessed the impact of communication apprehension on physician-patient communication, without looking specifically into the issue of sharing Internet search with physicians. Because heavy reliance on online health information has shown influence on physician patient interaction (Broom, 2005) and patients may concern about challenging physician authority if they share Internet search (Matusitz and Spear, 2015), it becomes important to examine if communication apprehension is related to sharing of online health information during doctor visits, and further affect physician-patient relationship.

Patients varying in their communication apprehension may feel different levels of comfort in sharing information with their physicians and differ in their directness of sharing Internet search. For instance, highly apprehensive patients are less willing to discuss online information with their physicians (Wheeless, 1984). As a result, they are more likely to secretly compare online information with physicians’ information without directly sharing it. In contrast, patients low on communication apprehension are less concerned about challenging physician authority and thus may engage with the information in more direct ways, such as directly asking questions or even presenting physicians with online information. Taken together, the following hypothesis is proposed:

H1: Patients’ communication apprehension will be negatively associated with directness of sharing online health information with their physicians.

## eHealth Literacy

eHealth literacy refers to individuals’ skills to effectively obtain, evaluate, and apply online information to health problems (Norman and Skinner, 2006). eHealth literacy has been found to be relevant to individuals’ health outcomes (Meherali et al., 2020). So far, much research has focused on the association between eHealth literacy and online health information seeking, a solitary behavior that requires minimal involvement of a communication partner (Chang et al., 2015); little attention has been devoted to the relationship between eHealth literacy and physician-patient interaction.

Past research examining the general health literacy sheds light on the association between eHealth literacy and physician-patient interaction (Katz et al., 2007; Hahn et al., 2015). Relevant studies found that patients with low health literacy tried to avoid situations that might show their limited understanding of health information and tended to report poor communication with their physicians (Sudore et al., 2009). For instance, Katz et al. (2007) found that low-literacy patients asked fewer questions than high-literacy patients during consultation. Diabetes patients with higher health literacy tend to speak more with their physicians to acquire relevant information (Hahn et al., 2015). Building upon past research which shows a positive relationship between health literacy and physician-patient interaction, it is expected that eHealth literacy will positively impact patients’ directness to share online health information with their physicians. Specifically, patients with low eHealth literacy may not feel confident to openly discuss online health information with their physicians. They may covertly compare online information with the information provided by physicians. In contrast, patients with high eHealth literacy are more assertive and willing to discuss online information with their physicians, thus using more direct ways to reveal such information. In fact, patients with high eHealth literacy reported to have presented the physician with the information they retrieved and asked significantly more questions than patients with low eHealth literacy (Neter and Brainin, 2012). Therefore, we assumed that:

H2: Patients’ eHealth literacy will be positively associated with directness of online health information sharing with their physicians.

## Physician Reactions and Patient Satisfaction

Although patients are concerned about physicians’ reactions and sometimes choose not to explicitly share Internet search with their physicians, patients who choose to reveal such information generally receive positive feedback from their physicians (Kivits, 2006). For instance, Sleath et al. (1999) found that physicians perceived question-asking in a positive way. Patients who asked questions were perceived to be more interested, but not more irritated than patients who did not ask questions by their physicians. Sommerhalder et al. (2009) found that physicians mostly appreciated their patients openly discuss online information with them, despite that contradictory information found online may sometimes cause conflict during consultation. AlGhamdi and Moussa (2012) reported that the majority of patients who discussed online information with their physicians believed the discussion positively affected their relationships with physicians. Taken together, most physicians tend to respond positively to patients who openly share online health information, albeit incidents of misunderstanding and conflicts. Therefore, it is hypothesized that using more direct ways of sharing online information tends to perceive more positive feedback from physicians.

H3: directness of online health information sharing will be positively related to patients’ perceived physician reactions.

Patients’ satisfaction has been recognized as an important assessment of health outcomes (Grogan et al., 2000). Patients’ satisfaction with their physicians has a significant impact on key health measures such as adherence to medicine and health status (Brown et al., 2003). In addition, patients’ satisfaction is closely related to physician-patient interaction (Street et al., 2009). Open and receptive communication tends to create a positive communicative atmosphere and leads to greater satisfaction from patients (Dutta-Bergman, 2005). Greene et al. (1994) found that physicians’ positive feedback, such as supportiveness to patients, leads to greater patient satisfaction. Therefore, it is expected that positive feedback from physicians can enhance patient satisfaction with their physicians.

H4: Perceived physician reactions will be positively associated with patient satisfaction.

The previous section examined the direct links between directness of online health information sharing and its antecedents as well as its relational outcomes. This study strived to take a step further to examine if mediation relationships would be discovered among the variables. Specifically, we wanted to examine if and how two patient characteristics—communication apprehension and eHealth literacy—would affect directness of online health information sharing, and thereby perceived physician reaction and patient satisfaction. Based on the rationale aforementioned, lower levels of communication apprehension is expected to facilitate direct sharing of online health information. Directness of sharing is hypothesized to be positively associated with perceived physician reactions, which is predicted of a positive relationship with patient satisfaction. Taken together, we hypothesize that:

H5: Patients with lower levels of communication apprehension will more directly share their Internet search with physicians, which will positively impact perceived physician reactions and thus patient satisfaction.

In contrast to communication apprehension, eHealth literacy is expected to be associated with directness of online sharing in the oppositive direction. Higher levels of eHealth literacy may lead to more direct sharing of online health information. The associations among directness of sharing, perceived physician reaction, and patient satisfaction are expected to be the same. Therefore, the following hypothesis is proposed.

H6: Patients with higher levels of eHealth literacy will more directly share their Internet search with physicians, which will positively impact perceived physician reactions and thus patient satisfaction.

**FIGURE 1:**
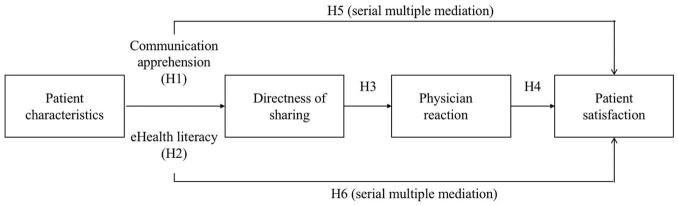
Schematic overview of the theoretical model and study hypotheses.

Put together, the current study integrates these proposed pathways into a comprehensive model shown in Figure 1.

## Materials and Methods

This study surveyed participants (above 18 years old) from China. Anyone who had ever visited a doctor could participated in this study. A total of 1,590 participants were recruited from a Chinese crowdsourcing platform Sojump^1^ and received a small amount of payment for their participation. Fifty-three participants (3.3%) failed one or more attention check questions in the survey and were deleted from final analyses, leaving a total of 1,537 valid cases (Female: 56.5%; Age: *M* = 30.68, *SD* = 7.84). Among 1,537 participants, 1,191 (77.5%) individuals reported to have searched for health information online before their doctor visits, and 818 (53.2%) individuals reported to have used online information during doctor visit. Because this study primarily concerned patients who used online health information during doctor visits, subsequent analyses were based on data collected from 818 participants.

Each participant was asked to fill out a questionnaire based on their most recent physician visits. Specifically, each participant was instructed to answer questions about their online health information seeking prior to their doctor visit, whether and how they reveal the information to their physicians, online health literacy, perceived physician reactions, and patient satisfaction with their physicians. Demographic information such as age, sex, health status, and education levels were also asked in the survey.

### Measures

#### Communication Apprehension

This trait was measured with a scale of four items modified from past research (Kim et al., 2000). The items were measured on a 5-point Likert scale (*1* = *strongly disagree*; *5* = *strongly agree*), showing a good reliability (*M* = 2.18, *SD* = 0.77, α = 0.88). Sample items include “[I] am not nervous when I have to talk to a physician.” and “Ordinarily, I am very tense and nervous when communicating with a physician.”

#### eHealth Literacy

A modified scale based on Norman and Skinner’s (2006) research was used to assess patients’ online health literacy. Seven questions on a 5-point Likert scale (*1* = *strongly disagree*; *5* = *strongly agree*) were asked. Sample items include “[I] know how to find helpful health resources on the Internet” and “I know what health resources are available on the Internet.” The scale reached satisfactory reliability (*M* = 3.86, *SD* = 0.55, α = 0.77).

#### Directness of Online Health Information Sharing With Physicians

Due to a lack of existing measures, this study generated the survey items based on quantitative research on this topic (Tan and Goonawardene, 2017). Each participant was asked to choose only one primary way that they used to share health information during their doctor visits, with four options ranging from the most indirect to the most direct way of sharing online health information with physicians (*1* = secretly compared online information with information provided by your physician; *2* = making suggestions to your physician based on online information, without explicitly mentioning the information was found online; *3* = explicitly told your physician that you searched for health information online and asked questions based on online information; *4* = directly showed online information to your physician.) Because these options differ in directness in a progressive manner, a higher score indicated more directness in information sharing. The average was calculated (*M* = 2.40; *SD* = 0.997).

#### Perceived Physicians’ Reactions

Five items based on Tan and Goonawardene’s (2017) work were used to assess how patients perceive physician reactions during doctor visits. The items were measured on a five-point Likert scale (*1* = strongly disagree; *5* = strongly agree). Sample items include “[T]he doctor was very receptive to online information that you revealed.” and “The doctor was open to discuss online information that you revealed.” The average scale score is 3.55 (*SD* = 0.75, α = 0.83).

#### Patient Satisfaction

To assess the extent to which patients are satisfied with their physicians, we used a scale of seven items modified from prior research (Loblaw et al., 1999; Grogan et al., 2000). All items (e.g., “[I] have absolute faith and confidence in my doctor”; “I will follow the doctor’s advice because I think he/she is absolutely right”) were measured on a 5-point Likert scale (1 = strongly disagree; 5 = strongly agree) and reached satisfactory reliability (*M* = 3.75, *SD* = 0.54, α = 0.81).

#### Control Variables

We included sex (*1* = *male*, *2* = *female*), age (by years), educational level, and self-perceived health status as control variables. Education level was assessed by asking about the obtained highest educational degree by five levels (as shown in Table 1). For self-perceived health status, participants were asked to rate their health on a 5-point Likert scale (*1* = *poor*; *5* = *excellent*). The average score of health status is 3.34 (*SD* = 0.74).

**TABLE 1:** Sample characteristics.

**Gender**	*n* (%)
Female, n (%)	474 (57.9)
Male	344 (42.1)
**Age group**	
18–25 y	213 (26.0)
26–35 y	446 (54.5)
36–45 y	121 (14.8)
46–55 y	31 (3.8)
>=56 y	2 (0.2)
**Education**	
Less than a high school diploma	1 (0.1)
High school degree	22 (2.7)
Associate degree	99 (12.1)
Bachelor’s degree	614 (75.1)
Master’s and doctorate degree	82 (10.0)

**Total *N***	818

### Analytical Approach

Descriptive statistics and zero-order correlations were conducted using SPSS 24.0. We used the PROCESS macro model 4 for SPSS for the single path mediation analysis, and model 6 for the serial mediation analysis (Hayes, 2017). The PROCESS macro estimates direct and indirect effects using 5,000 bootstrap samples. The results are presented as 95% bias correlated confidence intervals. When the confidence intervals do not contain zero, a significant indirect or mediating effect occurs. All control variables were included in the macro as covariates.

## Results

### Preliminary Analysis

Table 2 presented descriptive statistics and bivariate correlations among the variables. Compared to men, women indicated higher levels of communication apprehension [*t*(816) = −2.60, *p* = 0.009, *M*_men_ = 2.10, *SD*_*men*_ = 0.72, *M*_women_ = 2.24, *SD_*women*_* = 0.72], whereas women indicated lower levels of eHealth literacy [*t*(816) = 3.22, *p* < 0.001, *M*_*men*_ = 3.94, *SD*_*men*_ = 0.52, *M*_women_ = 3.81, *SD_*women*_* = 0.56]. Older people reported lower levels of communication apprehension (*r* = −0.16, *p* < 0.001) and higher levels of eHealth literacy than younger people (*r* = 0.21, *p* < 0.001). Those who indicated having poorer health status reported higher levels of communication apprehension (*r* = −0.14, *p* < 0.001), more positive physician reactions (*r* = 0.12, *p* < 0.001), higher levels of patient satisfaction (*r* = 0.18, *p* < 0.001), but lower levels of eHealth literacy(*r* = −0.09, *p* = 0.009).

**TABLE 2:** Mean, standard deviation, and zero-order correlations (*N* = 818).

Variable	M	SD	1	2	3	4	5	6	7	8
1. Communication apprehension	2.18	0.77	–							
2. Online health literacy	3.87	0.55	−0.28***	–						
3. DOHISP	2.40	1.00	−0.10**	0.06	–					
4. Perceived physicians’ reactions	3.55	0.75	−0.32***	0.30***	0.15***	–				
5. Patient satisfaction	3.75	0.54	−0.28***	0.28***	0.11**	0.54***	–			
6. Sex	–	–	0.09**	−0.11**	–0.03	–0.02	0.00	–		
7. Age	30.26	7.02	−0.16***	0.21***	–0.03	–0.03	0.06	−0.08*	–	
8. Education level	5.92	0.58	–0.04	0.03	–0.01	–0.00	–0.01	0.04	−0.09*	–
9. Health status	3.34	0.74	−0.14***	0.09**	0.03	0.12***	0.18***	–0.01	–0.05	0.10**

DOHISP, Directness of online health information sharing with physicians. *p < 0.05; **p < 0.01; ***p < 0.001.

Communication apprehension was negatively related to directness of sharing online health information with their physicians (*r* = −0.10, *p* = 0.004). Directness of sharing online health information with their physicians was positively related to perceived physicians’ reactions (*r* = 0.15, *p* < 0.001) and with patient satisfaction (*r* = 0.11, *p* = 0.001). Additionally, perceived physicians’ reactions were positively related to patient satisfaction (*r* = 0.54, *p* < 0.001). These correlations provided some initial evidence for mediating chain among communication apprehension, directness of sharing online health information with their physicians, perceived physicians’ reactions, and patient satisfaction. However, contrary to our expectation, the correlation between patients’ eHealth literacy was not significantly related to directness of sharing online health information with their physicians (*r* = 0.06, *p* = 0.075). Therefore, further mediation analysis was only conducted on the model with communication apprehension.

### Direct Relationships

Communication apprehension was modeled as the predictor and patient satisfaction was the dependent variable. Directness of sharing online health information with their physicians and perceived physicians’ reactions were the first and second mediator, respectively. Sex, age, educational level, and health status were included as covariates because preliminary analysis showed meaningful correlation patterns among covariates and variables of interest.

The first hypothesis proposed a negative association between communication apprehension and directness of sharing online health information with their physicians (H1). Results confirmed the negative relationship (*b* = −0.136, *se* = 0.047, *p* = 0.004, *95% CI* [−0.227, −0.044]). Due to the non-significant correlation between online information literacy and directness of sharing online health information with their physicians (*r* = 0.06, *p* = 0.075), H2 was not supported. We then proposed a positive association between directness of online health information sharing and perceived physicians’ reactions (H3). The results also supported this relationship (*b* = 0.092, *se* = 0.025, *p* < 0.001, *95% CI* [0.043, 0.141]). In addition, a positive association between perceived physicians’ reactions and patient satisfaction was proposed (H4). Supporting H4, the results found a significant positive relationship (*b* = 0.353, *se* = 0.023, *p* < 0.001, *95% CI* [0.308, 0.397]).

### Indirect Relationships

**FIGURE 2:**
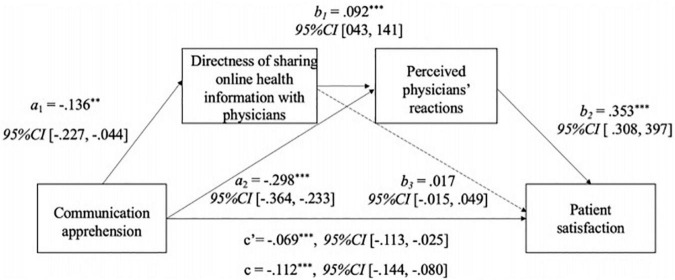
The results for serial multiple mediation model with communication apprehension. Analyses are based on 5,000 bootstrap samples, controlling for sex, age, education level, and health status. Path coefficients are unstandardized coefficient. → significant paths; ⇢ non-significant paths. Indirect effect (a_1_b_1_b_2_): effect size = −0.004, Boot *SE* = 002, 95% CI [−0.009, −0.001]. Indirect effect (a_1_b_3_): effect size = −0.002, Boot *SE* = 003, 95% CI [−0.009, 0.002]. Indirect effect (a_2_b_2_): effect size = −0.105, Boot *SE* = 016, 95% CI [−0.137, −0.074]. ^**^p < 0.01; ^***^p < 0.001.

H5 proposed a serial mediation model among communication apprehension, directness of sharing online health information with their physicians, perceived physician reactions, and patient satisfaction (see Figure 2). Supporting H5, patients’ communication apprehension was found to negatively affect the directness to share Internet search with their physicians (*a*_1_ = −0.136, *p* = 0.004). Directness of sharing online information with physicians then positively affected patients’ perceived physician reactions (b_1_ = 0.092, *p* < 0.001), which in turn, led to a positive impact on patient satisfaction (b_2_ = 0.353, *p* < 0.001). The mediation was confirmed by a 5,000 bootstrapping analysis (effect size = −0.004, Boot *SE* = 0.002, *95% CI* [−0.009, −0.001]). H6 was not analyzed because the association between online information literacy and directness of sharing online health information with their physicians was not significant.

## Discussion

While becoming more informed with Internet search, patients may also debate if online health information should be shared with their physicians and in what ways. This study contributes to a more comprehensive understanding of online health information sharing through an empirical assessment of the associations among patients’ communication characteristics, directness of information sharing, and physician-patient relationship. Supporting the hypotheses, the results showed that less apprehensive patients used more direct ways to share online health information with their physicians (H1), which in turn positively affected perceived reactions from physicians and patient satisfaction (H3–H5). The findings are line with previous research that suggested communication apprehension is a key factor that influences patients’ directness of sharing online search with their physicians (Perrault and Silk, 2015).

In contrast with H2, results showed patients’ eHealth literacy showed no association with directness of online health information sharing. As such, the serial multiple mediation hypothesis with eHealth literacy was also not supported (H6). Although prior research suggests that a higher level of health literacy tend to motivate more open discussion of health information with physicians (Katz et al., 2007), it is possible that people with higher eHealth literacy feel more competent in evaluating online health information and spotting misinformation (Diviani et al., 2015). Therefore, they may not feel necessary to directly discuss the information with their physicians. Given that this is the first known study that investigated the relationship between eHealth literacy and directness of information sharing with physicians, future research can look into the relationships by examining the possible competing underlying mechanisms mentioned above.

### Theoretical and Practical Implications

From the perspective of patients, this study develops a preliminary model of online health information sharing. This model presents a process of health communication by taking into account antecedents that motivate online health information sharing and relational outcomes affected by this construct. The model extends previous research that differentiates various ways of online health information sharing by focusing on the dimension of directness in information sharing. Online health information sharing, as a multifaceted construct, can be explored along a variety of dimensions (e.g., frequency, directness). Among the underexplored dimensions of online health information sharing, directness is perhaps one of the most prominent dimensions affecting the communicative process of physician-patient interaction. Directly sharing Internet search with physicians demonstrates patients’ sense of control in the medical system (Tan and Goonawardene, 2017). In an era of health consumerism, an emphasis on patient empowerment can facilitate positive communication between patients and physicians, which may further bring optimal outcomes in treatment (Brown et al., 2003).

This study offers some practical implications for sharing online health information with physicians as well as improving physician-patient interaction. Given that health information acquired online may be inaccurate and misleading (Scherer et al., 2021), it is imperative to encourage Internet-informed patients to discuss Internet search with their physicians. This study showed some promising results that could motivate patients to directly share and discuss online health information with their physicians. Past research suggested that a major concern that discouraged patients from sharing online health information with their physicians was physicians’ negative feedback (Silver, 2015). This study, however, showed that patients who choose to openly discuss such information tend to perceive positive feedback from their physicians and increase patient satisfaction. Based on the encouraging results revealed in this study, it is necessary to educate patients to not only search for health information online, but more importantly directly share the information with their physicians, rather than covertly comparing such information with the information provided by physicians.

In order to facilitate more open discussion with physicians and thus improve physician-patient relationship, health professionals and organizations can make an effort to reduce patients’ communication apprehension while visiting doctors. For instance, Perrault and Silk (2015) suggested that providing information such as physician biographies to patients prior to their doctor visit can help patients reduce uncertainty toward prospective physicians and ease their communication apprehension during their visit. Besides providing additional information to patients, practitioners can explore alternatives that may reduce patients’ communication apprehension and promote more effective physician-patient interaction. For example, supportive attitudes from physicians may help patients to feel less nervous to discuss Internet search. Situational factors such as having a company during a doctor visit may ease a patient’s communication apprehension. However, we should be aware that some patients may become cyberchondria and obsessed with online health information-seeking (Zheng et al., 2021). This type of patients may ask physicians endless questions to seek for reassurance. Their sharing of Internet search, if excessively, may not be welcomed by physicians. Future research can try to test the boundaries of information sharing frequency and physician-patient interaction outcomes.

### Limitations and Future Research

This study has several limitations that point to directions for future research. First, data were collected through a cross-sectional survey and thus may limit our ability to make causal claims between online health information sharing and relational outcomes. We have tried to eliminate this concern by considering time sequence in question-asking. For example, perceived physician reactions toward information sharing have to take place after patients shared Internet search with them. In addition, eHealth literacy and communication apprehension as patients’ characteristics, were more reasonably treated as antecedents rather than outcome variables. In the future, research could strive to conduct longitudinal surveys or experiments to closely examine the causal effects between online health information sharing and physician-patient relationship.

Second, data were collected through an online crowdsourcing platform and thus may not match the demographic characteristics of the entire population. For instance, the majority of the sample were below the age of 60, making it difficult to generalize our findings to the elderly population. The low proportion of elderly participants is partially due to the low accessibility of this population on the recruiting platform. In addition, elderly people are less active in online health information seeking (Bennett et al., 2009; Jacobs et al., 2017), leaving a small size of eligible sample to participate in this study. Future research may target specifically the elderly population and examine their online health information seeking and sharing behaviors.

Third, how patients share online health information with their physicians can be affected by many factors, not limited to the two patient characteristics examined in this study. For instance, participants varying in cultural backgrounds could differ in their sharing behaviors. This study used Chinese participants who are embraced by a culture with high uncertainty avoidance. As a result, these participants tend to be less straightforward in sharing online health information with their physicians compared with those from a culture with low uncertainty avoidance. It would be interesting to compare patterns of health information sharing across cultures. Further, online health information sharing can be mutually influenced by contextual factors, patient characteristics, and physician characteristics (Tan and Goonawardene, 2017). It is meaningful to investigate how different factors work together to achieve a comprehensive understanding of the communication process. In addition, this study only examined physician-patient interaction as the outcome variable. Future research should examine how online health information sharing with physicians may affect patients’ health outcomes.

## Data Availability Statement

The raw data supporting the conclusions of this article will be made available by the authors, without undue reservation.

## Ethics Statement

The studies involving human participants were reviewed and approved by Zhejiang University. Written informed consent for participation was not required for this study in accordance with the national legislation and the institutional requirements.

## Author Contributions

SL was in charge of survey design, data collection, and manuscript writing. KW was in charge of data analysis and manuscript writing. All authors contributed to the article and approved the submitted version.

## Conflict of Interest

The authors declare that the research was conducted in the absence of any commercial or financial relationships that could be construed as a potential conflict of interest.

## Publisher’s Note

All claims expressed in this article are solely those of the authors and do not necessarily represent those of their affiliated organizations, or those of the publisher, the editors and the reviewers. Any product that may be evaluated in this article, or claim that may be made by its manufacturer, is not guaranteed or endorsed by the publisher.

## Footnotes

1
http://www.wjx.cn

## References

[B1] AlGhamdi K. M. Moussa N. A. (2012). Internet use by the public to search for health-related information. *Int. J. Med. Inf.* 81 363–373. 10.1016/j.ijmedinf.2011.12.004 22217800

[B2] Bennett J. A. Cameron L. D. Whitehead L. C. Porter D. (2009). Differences between older and younger cancer survivors in seeking cancer information and using complementary/alternative medicine. *J. Gen. Intern. Med.* 24 1089–1094. 10.1007/s11606-009-0979-8 19685099 PMC2762516

[B3] Booth-Butterfield S. Chory R. Beynon W. (1997). Communication apprehension and health communication and behaviors. *Commun. Q.* 45 235–250.

[B4] Briones R. (2015). Harnessing the web: how e-Health and e-Health literacy impact young adults’ perceptions of online health information. *Med. 2 0* 4:e5. 26721292 10.2196/med20.4327 PMC4713906

[B5] Broom A. (2005). Virtually he@lthy: the impact of internet use on disease experience and the doctor-patient relationship. *Qual. Health Res.* 15 325–345. 10.1177/1049732304272916 15761103

[B6] Brown J. Stewart M. Ryan B. (2003). “Outcomes of patient–provider interaction,” in *Handbook of Health Communication*, eds Thompson T. L. Dorsey A. M. Miller K. I. Parrott R. (Mahwah, NJ: Lawrence Erlbaum Associates, Inc.), 141–161.

[B7] Chang F. C. Chiu C. H. Chen P. H. Miao N. F. Lee C. M. Chiang J. T. (2015). Relationship between parental and adolescent eHealth literacy and online health information seeking in Taiwan. *Cyberpsychol. Behav. Soc. Netw.* 18 618–624. 10.1089/cyber.2015.0110 26375050

[B8] Diviani N. van den Putte B. Giani S. van Weert J. C. (2015). Low health literacy and evaluation of online health information: a systematic review of the literature. *J. Med. Internet Res.* 17:e112. 10.2196/jmir.4018 25953147 PMC4468598

[B9] Dutta-Bergman M. J. (2005). The relation between health-orientation, provider-patient communication, and satisfaction: an individual-difference approach. *Health Commun.* 18 291–303. 16187933 10.1207/s15327027hc1803_6

[B10] Greene M. G. Adelman R. D. Friedman E. Charon R. (1994). Older patient satisfaction with communication during an initial medical encounter. *Soc. Sci. Med.* 38 1279–1288. 8016691 10.1016/0277-9536(94)90191-0

[B11] Grogan S. Conner M. Norman P. Willits D. Porter I. (2000). Validation of a questionnaire measuring patient satisfaction with general practitioner services. *Qual. Health Care* 9 210–215. 11101705 10.1136/qhc.9.4.210 PMC1743536

[B12] Hahn E. A. Burns J. L. Jacobs E. A. Ganschow P. S. Garcia S. F. Rutsohn J. P. (2015). Health literacy and patient-reported outcomes: a cross-sectional study of underserved English- and Spanish-speaking patients with yype 2 diabetes. *J. Health Commun.* 20(Suppl. 2) 4–15. 10.1080/10810730.2015.1061071 26513026

[B13] Hart A. Henwood F. Wyatt S. (2004). The role of the Internet in patient-practitioner relationships: findings from a qualitative research study. *J. Med. Internet Res.* 6:e36. 10.2196/jmir.6.3.e36 15471762 PMC1550614

[B14] Hayes A. F. (2017). *Introduction to Mediation, Moderation, and Conditional Process Analysis: A Regression-Based Approach.* New York, NY: Guilford publications.

[B15] Hu X. Bell R. A. Kravitz R. L. Orrange S. (2012). The prepared patient: information seeking of online support group members before their medical appointments. *J. Health Commun. Int. Perspect.* 17 960–978. 10.1080/10810730.2011.650828 22574697

[B16] Jacobs W. Amuta A. O. Jeon K. H. (2017). Health information seeking in the digital age: an analysis of health information seeking behavior among US adults. *Cogent Soc. Sci.* 3 1–11. 10.1080/23311886.2017.1302785

[B17] Katz M. G. Jacobson T. A. Veledar E. Kripalani S. (2007). Patient literacy and question-asking behavior during the medical encounter: a mixed-methods analysis. *J. Gen. Intern. Med.* 22 782–786. 10.1007/s11606-007-0184-6 17431697 PMC2583801

[B18] Kim M. S. Klingle R. S. Sharkey W. F. Park H. S. Smith D. H. Cai D. (2000). A test of a cultural model of patients’ motivation for verbal communication in patient-doctor interactions. *Commun. Monogr.* 67 262–283. 10.1080/03637750009376510

[B19] Kivits J. (2006). Informed patients and the internet: a mediated context for consultations with health professionals. *J. Health Psychol.* 11 269–282. 10.1177/1359105306061186 16464924

[B20] Loblaw D. A. Bezjak A. Bunston T. (1999). Development and testing of a visit-specific patient satisfaction questionnaire: the Princess Margaret Hospital Satisfaction With Doctor Questionnaire. *J. Clin. Oncol.* 17 1931–1938. 10.1200/JCO.1999.17.6.1931 10561235

[B21] Matusitz J. Spear J. (2015). Doctor-patient communication styles: a comparison between the United States and three Asian countries. *J. Hum. Behav. Soc. Environ.* 25 871–884. 10.1080/10911359.2015.1035148

[B22] McCroskey J. C. (1984). “The communication apprehension perspective,” in *Avoiding Communication: Shyness, Reticence, and Communication*, eds Daly J. A. McCroskey J. C. (Beverly Hills, CA: SAGE Publications), 13–38.

[B23] Meherali S. Punjani N. S. Mevawala A. (2020). Health literacy interventions to improve health outcomes in low- and middle-income countries. *Health Lit. Res. Pract.* 4 e251–e266. 10.3928/24748307-20201118-01 33313935 PMC7751448

[B24] Neter E. Brainin E. (2012). eHealth literacy: extending the digital divide to the realm of health information. *J. Med. Internet Res.* 14:e19. 22357448 10.2196/jmir.1619 PMC3374546

[B25] Norman C. D. Skinner H. A. (2006). eHEALS: the eHealth literacy scale. *J. Med. Internet Res.* 8:e27. 10.2196/jmir.8.4.e27 17213046 PMC1794004

[B26] Perrault E. K. Silk K. J. (2015). Reducing communication apprehension for new patients through information found within physicians’ biographies. *J. Health Commun.* 20 743–750. 10.1080/10810730.2015.1018569 25942070

[B27] Scherer L. D. McPhetres J. Pennycook G. Kempe A. Allen L. A. Knoepke C. E. (2021). Who is susceptible to online health misinformation? A test of four psychosocial hypotheses. *Health Psychol.* 40 274–284. 10.1037/hea0000978 33646806

[B28] Silver M. P. (2015). Patient perspectives on online health information and communication with doctors: a qualitative study of patients 50 years old and over. *J. Med. Internet Res.* 17:e19. 10.2196/jmir.3588 25586865 PMC4319073

[B29] Sleath B. Roter D. Chewning B. Svarstad B. (1999). Asking questions about medication: analysis of physician-patient interactions and physician perceptions. *Med. Care* 37 1169–1173. 10.1097/00005650-199911000-00009 10549619

[B30] Sommerhalder K. Abraham A. Zufferey M. C. Barth J. Abel T. (2009). Internet information and medical consultations: experiences from patients’ and physicians’ perspectives. *Patient Educ. Couns.* 77 266–271. 19411157 10.1016/j.pec.2009.03.028

[B31] Stevenson F. A. Kerr C. Murray E. Nazareth I. (2007). Information from the Internet and the doctor-patient relationship: the patient perspective–a qualitative study. *BMC Fam. Pract.* 8:47. 10.1186/1471-2296-8-47 17705836 PMC2041946

[B32] Street R. L. Jr. Makoul G. Arora N. K. Epstein R. M. (2009). How does communication heal? Pathways linking clinician-patient communication to health outcomes. *Patient Educ. Couns.* 74 295–301. 10.1016/j.pec.2008.11.015 19150199

[B33] Suarez-Lledo V. Alvarez-Galvez J. (2021). Prevalence of health misinformation on social media: systematic review. *J. Med. Internet Res.* 23:e17187. 10.2196/17187 33470931 PMC7857950

[B34] Sudore R. L. Landefeld C. S. Pérez-Stable E. J. Bibbins-Domingo K. Williams B. A. Schillinger D. (2009). Unraveling the relationship between literacy, language proficiency, and patient–physician communication. *Patient Educ. Couns.* 75 398–402. 19442478 10.1016/j.pec.2009.02.019 PMC4068007

[B35] Tan S. S. Goonawardene N. (2017). Internet health information seeking and the patient-physician relationship: a systematic review. *J. Med. Internet Res.* 19:e9. 10.2196/jmir.5729 28104579 PMC5290294

[B36] Wang X. Shi J. Kong H. (2020). Online health information seeking: a review and meta-analysis. *Health Commun.* 36 1163–1175. 10.1080/10410236.2020.1748829 32290679

[B37] Wheeless V. E. (1984). Communication apprehension and trust as predictors of willingness to discuss gynecological health topics. *Commun. Res. Rep.* 1 117–121.

[B38] Wong D. K.-K. Cheung M.-K. (2019). Online health information seeking and eHealth literacy among patients attending a primary care clinic in Hong Kong: a cross-sectional survey. *J. Med. Internet Res.* 21:e10831. 10.2196/10831 30916666 PMC6456826

[B39] Zheng H. Kim H. K. Sin S. C. J. Theng Y. L. (2021). A theoretical model of cyberchondria development: antecedents and intermediate processes. *Telemat. Inf.* 63:101659.

